# From antiquity to contemporary times: how olive oil by-products and waste water can contribute to health

**DOI:** 10.3389/fnut.2023.1254947

**Published:** 2023-10-16

**Authors:** Adriana Albini, Francesca Albini, Paola Corradino, Laura Dugo, Luana Calabrone, Douglas M. Noonan

**Affiliations:** ^1^Istituto di Ricovero e Cura a Carattere Scientifico (IRCCS), European Institute of Oncology IEO, Milan, Italy; ^2^Royal Society for the Encouragement of Arts, Manufactures and Commerce, London, United Kingdom; ^3^Department of Science and Technology for Sustainable Development and One Health, University Campus Bio-Medico of Rome, Roma, Italy; ^4^I.S.B.-Ion Source and Biotechnologies, Milano, Italy; ^5^Department of Biotechnology and Life Sciences, University of Insubria, Varese, Italy; ^6^IRCCS MultiMedica, Milan, Italy

**Keywords:** olive oil, olive mill waste water, polyphenols, cancer prevention, cardiotoxicity, angiogenesis, health

## Abstract

Since antiquity, numerous advantages of olive oil and its by-products have been recognized in various domains, including cooking, skincare, and healthcare. Extra virgin olive oil is a crucial component of the Mediterranean diet; several of its compounds exert antioxidant, anti-proliferative, anti-angiogenic and pro-apoptotic effects against a variety of cancers, and also affect cellular metabolism, targeting cancer cells through their metabolic derangements. Numerous olive tree parts, including leaves, can contribute metabolites useful to human health. Olive mill waste water (OMWW), a dark and pungent liquid residue produced in vast amounts during olive oil extraction, contains high organic matter concentrations that may seriously contaminate the soil and surrounding waters if not managed properly. However, OMWW is a rich source of phytochemicals with various health benefits. In ancient Rome, the farmers would employ what was known as amurca, a mulch-like by-product of olive oil production, for many purposes and applications. Several studies have investigated anti-angiogenic and chemopreventive activities of OMWW extracts. The most prevalent polyphenol in OMWW extracts is hydroxytyrosol (HT). Verbascoside and oleuperin are also abundant. We assessed the impact of one such extract, A009, on endothelial cells (HUVEC) and cancer cells. A009 was anti-angiogenic in several *in vitro* assays (growth, migration, adhesion) and inhibited angiogenesis *in vivo*, outperforming HT alone. A009 inhibited cells from several tumors *in vitro* and *in vivo* and showed potential cardioprotective effects mitigating cardiotoxicity induced by chemotherapy drugs, commonly used in cancer treatment, and reducing up-regulation of pro-inflammatory markers in cardiomyocytes. Extracts from OMWW and other olive by-products have been evaluated for biological activities by various international research teams. The results obtained make them promising candidates for further development as nutraceutical and cosmeceutical agents or dietary supplement, especially in cancer prevention or even in co-treatments with anti-cancer drugs. Furthermore, their potential to offer cardioprotective benefits opens up avenues for application in the field of cardio-oncology.

## Historical background

The domestication of the olive tree started in the Mediterranean region many thousands of years ago, and the production of oil from its fruit could date back as far as 2,500 BCE (1, 2). Although olives were appreciated on their own as a staple food, oil extracted from the fruit was possibly the main reason why the olive tree became so largely cultivated. Apart from its employment at the dining table, both for cooking and condiment, olive oil was used for many other purposes in ancient times, namely as lamp fuel, personal grooming, cosmetics, soap, and medicine. Perhaps because of this versatility, coupled with its extraordinary longevity (olive trees can live 3,000+ years, see the famous “olive tree of Vouves” in Crete), it also acquired a religious and symbolic role. While not so much employed for food, olive oil was used in Egypt as early as the New Kingdom period (1550–1,070 BCE) for some of the above-mentioned purposes, such as for lighting and as an ingredient in cosmetics, but it was also an offering to the gods. The Minoans used olive oil in religious ceremonies too. The oil became a principal product of the Minoan civilization, where it is thought to have represented wealth (3). It was used in the anointing of priests and in the preparation of offerings for the gods. Olive oil was also present in Minoan funerary practices, where it was believed to help the soul of the deceased on their journey to the afterlife.

In Ancient Greece, the olive tree was considered sacred and a symbol of peace, prosperity, and wisdom. According to Greek mythology, Athena, the goddess of wisdom and warfare, competed against Poseidon, the god of the sea, for the patronage of the city of Athens. As part of the competition, they were both asked to present a gift to the city that would be of the greatest benefit to its people. Athena won by planting an olive tree on the Acropolis, which was not only beautiful, but also provided a valuable source of food, oil, and fuel for the city’s inhabitants. The Sacred Olive Tree can still be found in the Acropolis of Athens today. Of course, it is not the “original” one, but legend has it that it is a direct descendant, grown from propagation. The Olympic flame was lit using a concave mirror to focus the sun’s rays, and a little olive oil, while a wreath made of twisted olive branches crowned victors in the athletic competitions. Amphoras filled with olive oil were among the prizes for the winners (4). Olive wreaths were common in Rome as well, as a symbol of victory in military campaigns, but also to announce the birth of a baby boy. An olive branch, of course, is found in the Bible (Genesis 8:11) as a symbol of peace, brought back to Noah’s ark by a dove, and the recipe of the holy oil used by Moses to anoint priests and prophets includes olive oil in its ingredients. The olive tree and its fruits are also mentioned several times in the Quran. It is a symbol of strength, beauty, and prosperity, and is associated with blessings and divine guidance.

The curative powers of olive oil were already known in Ancient Egypt. The Ebers Papyrus is a medical text dating back to around 1,550 BCE. It contains recipes and remedies for various ailments, many of which involve the use of oils and ointments made from plants and other natural materials, including olive oil. There are recipes for ointments made with olive oil to treat skin conditions such as eczema and psoriasis, as well as to soothe insect bites and stings. It is also mentioned as a treatment for joint pain and eye infections. Even the Greeks believed that olive oil had medicinal properties, and it was recommended to treat skin conditions, digestive disorders and more. A mixture of olive oil combined with other oils was also used by Greek women as a form of birth control (5). Olive oil as a remedy for a number of ailments can be found in Traditional Persian Medicine (TPM). The Canon of Medicine (Al-Qanun fi al-Tibb), written by Avicenna (Ibn Sina) in the 11th century, mentions the use of olive oil for digestion, respiratory problems, skin diseases, joint pains, fevers, but also for mental health. Avicenna believed that olive oil had a calming effect on the nervous system and could be used to treat anxiety and depression. The Canon of Medicine is in many ways a synthesis of the medical knowledge of the ancient Greeks and the medical practices of the Islamic world. It drew extensively from the works of Hippocrates, Galen, and other ancient Greek physicians, as well as from the medical traditions of Persia, India, and other regions.

## Review methodology

The research strategy included the definition of keywords and a search of online databases: Scopus, Web of Science, PubMed and Google Scholar. When sources were chosen in accordance with the criteria, they had to be available online in one of the listed databases. To provide distinct results for the same collection of terms, we chose to utilise keyword sequences in search engines.

The following word groups were used for the search as Boolean string: olive*[TI] AND (by-product* OR byproduct* OR wastewater* OR waste*) AND (Health OR cancer OR prevention OR cells).

The literature used in this review was found considering “All year” in the search criteria and was manually chosen for relevance to the Research Topic.

We accepted all articles written in English and we excluded book reviews, editorials, commentaries, opinion pieces, and topic overviews that did not explicitly identify as literature reviews.

### Extra virgin oil production and waste water

Olive oil production normally encompasses several sequential stages, starting with harvesting and followed by cleaning and washing to eliminate leaves, twigs, dirt, and lingering impurities. The cleaned olives are then subjected to grinding, where they are crushed or ground to form a paste. Subsequently, the olive paste undergoes malaxation, a process where it is thoroughly mixed to facilitate the breakdown of oil droplets and the release of oil. After malaxation, the oil is separated from the paste either through a press or a centrifuge. Following this separation, the oil undergoes filtering to eliminate any remaining particles or impurities. Lastly, to preserve its flavor and quality, the oil is stored in a cool, dark environment. Extra virgin olive oil (EVOO) is a type of olive oil that is obtained mechanically, without the use of heat or chemicals, from olive fruits. It is considered the highest quality and most flavorful type of olive oil and is widely used as a condiment. EVOO has a rich, fruity taste and is high in monounsaturated fatty acids (MUFA), which are considered beneficial for heart health. It is also a good source of antioxidants, including polyphenols and vitamin E, which may help protect against oxidative damage in the body. Together with a wide variety of seasonal fruits, vegetables, whole grains, legumes, fish, nuts, and moderate amounts of cheese, meat, and wine, olive oil is an integral part of the Mediterranean diet (MD). This diet has been shown to have a range of health benefits, including a reduced risk of cardiovascular pathologies, type 2 diabetes, neurodegenerative diseases, and some cancers (6, 7).

Olive trees provide numerous products and by-products rich in biologically active molecules (8). During the washing and processing of the olive fruit, large quantities of a dark, odorous effluent, known as OMWW, are generated. Interestingly, analyses of bioactive compounds distribution throughout the entire EVOO mill production chain evidenced the abundance of molecules in by-products such as OMWW and pomace, although the content is highly variable due to the characteristics of the olives and to the sample preparation steps used (9). The high concentration of organic matter contained in OMWW, which includes tannins, polyphenols, polyalcohols, proteins, organic acids, pectins, and lipids, may seriously damage the environment if not managed properly. It can contaminate the surrounding soil and water resources. It can also cause eutrophication, leading to increased plant and algae growth. Mediterranean regions produce about 97% of the worldwide olive oils, recently, olive tree products are increasing (30 million tons), approximately 5 kg of olives are needed to obtain 1 liter of oil; OMWW has been estimated at around 20 million m^3^, and 1 m^3^ of OMWW corresponds to 100–200 m^3^ of domestic sewage (10–12). These figures can give us an idea of the extent of the problem. Many countries have been led to develop new technologies to deal with the issue to reduce pollution (13, 14). In recent years, intensive research in the field of OMWW management has suggested that these effluents may be a very valuable resource of chemical substances for a variety of purposes, from medicine to agriculture.

In Roman times the production of olive oil was already in the order of tens of millions of liters per year, so it was necessary to find ways to dispose of waste water (15). Simply dumping it into nearby rivers or lakes had serious environmental consequences, leading to water pollution and harm to aquatic ecosystems. A less environmentally damaging solution was to collect the OMWW in underground pits or pits lined with impermeable materials, such as clay, in order to allow the water to slowly evaporate or seep into the ground. This method was not yet ideal, as over time it could lead to soil and groundwater contamination. But not all the OMWW ended up being discarded. The dark, smelly, and bitter tasting water produced during the oil extraction process was known by the Mediterranean populations to have many useful qualities. Called amorge by the Greeks and amurca by the Romans, it had a number of applications (15, 16). It could be used as feed for animals, as fertilizer or pesticide for insects and weed, as lubricant for axles and belts (17–19). It was also employed in the making of plaster (20). In medicine, it was prescribed as a drink for various ailments, including gastrointestinal problems such as indigestion, constipation, and diarrhea. It was also used as a topical treatment for skin conditions like wounds, burns, and insect bites. Amurca was believed to have antiseptic and anti-inflammatory properties, which made it useful in treating these conditions (21).

**Figure 1:**
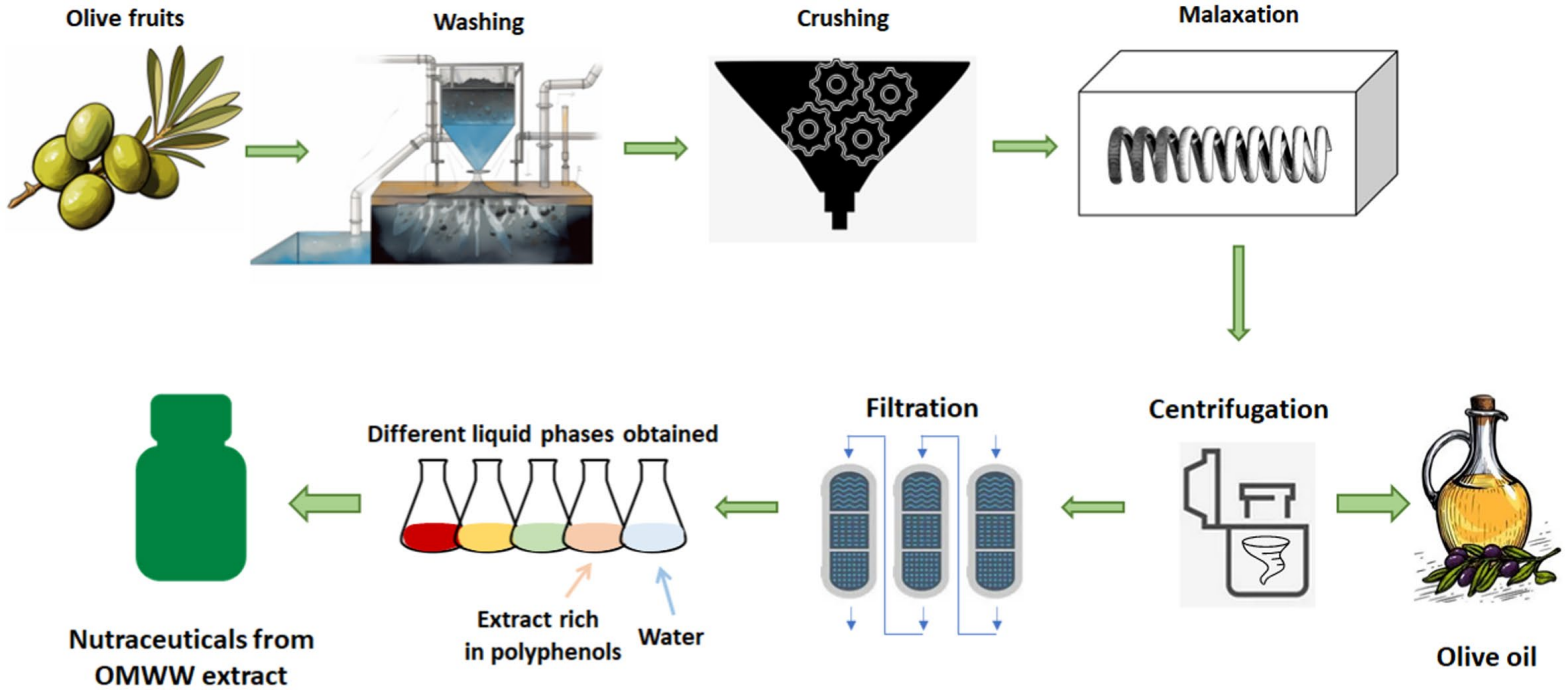
Olive oil production and valorization of olive mill wastewater products.

The tradition of drinking OMWW for health reasons among farmers has continued almost to the present day, thus arousing the interest of both oil producers and scientific researchers (Figure 1).

What makes this by-product of olive oil production so powerful? OMWW contains a concentration of phytochemicals that is at least tenfold that of EVOO (22). Phytochemicals have been linked to various health benefits, such as reducing the risk of chronic diseases, including cancer, cardiovascular disease, and diabetes (Figure 1).

OMWW’s exact composition can vary depending on factors such as olive variety, extraction process, and treatment methods, but usually includes:

- Phenolic compounds: aromatic compounds that can be found in various concentrations. They are responsible for the bitter taste of olive oil and have antioxidant properties.
- Fatty acids: organic acids that are found in high concentrations. They are the primary constituents of olive oil and are responsible for its characteristic taste and aroma.
- Carbohydrates: compounds that are found mainly in the form of sugars and are the primary source of energy for the microorganisms that degrade OMWW.
- Nitrogen-containing compounds: organic compounds that contain nitrogen atoms. They mainly occur in the form of proteins and amino acids, which are essential for the growth of microorganisms.
- organic acids: organic compounds characterized by acidic properties, which are found in varying concentrations in OMWW and are responsible for its low pH.

Polyphenols are a group of chemical substances found in plants. Their consumption may play an important role in maintaining health through the regulation of metabolism, obesity, chronic disease, and cell growth. OMWW contains at least 30 types of polyphenols in high concentration, among which (23–25):

- Hydroxytyrosol (HT): one of the most abundant polyphenols in OMWW. It is a potent antioxidant that has been shown to have anti-inflammatory, cardioprotective, and neuroprotective properties.
- Tyrosol (TYR): a phenolic compound with antioxidant, anti-inflammatory, and anti-microbial properties, precursor of HT.
- Oleuropein: a glycosylated secoiridoid with antioxidant, anti-inflammatory, and anti-microbial properties.
- Verbascoside: a phenylpropanoid glycoside with antioxidant, anti-inflammatory, and neuroprotective properties.
- Ligstroside: a polyphenol with antioxidant and anti-inflammatory effects and which may also support cardiovascular health.
- Luteolin: a flavone that has been shown to have antioxidant, anti-inflammatory, and anti-cancer properties.

OMWW can also contain vanillic acid, caffeic acid, p-coumaric acid, chlorogenic acid, ferulic acid and elenolic acid (23–26).

Treatment of waste water for use in various applications could contribute to sustainable water consumption and ecosystem conservation. Modern technology is making the process of extraction (23–26) much more efficient and economically and environmentally viable than in the past. This, paired with the increasing need to reduce and utilize agricultural waste, means that OMWW has now garnered much interest for its potential applications in many different industries (27). There are several new extraction processes that are being used, including:

- Membrane filtration: this process uses semi-permeable membranes to separate the different components of OMWW, such as water, organic acids, and polyphenols, based on their molecular weight and size.
- Liquid–liquid extraction: this method involves using solvents to selectively extract specific compounds from OMWW, such as polyphenols, which can then be further processed and purified.
- Enzymatic hydrolysis: this process uses enzymes to break down complex molecules into simpler, more valuable compounds, such as glucose or fructose, which can be used in various industries.
- Supercritical fluid extraction: this method uses high-pressure and temperature conditions to extract compounds from OMWW, such as polyphenols and organic acids, using a supercritical fluid, such as carbon dioxide.
- Adsorption: this process involves the use of adsorbent materials, such as activated carbon, to selectively remove specific compounds from OMWW, such as polyphenols, while leaving other components behind (27).

Through the various processes, OMWW can find applications in several different fields. In agriculture it can be used as an irrigation source for non-food crops and as fertilizer, due to its high organic matter content. It can have industrial use in the production of biodegradable plastics, surfactants, and biofuels. It can be utilized as a source of energy through anaerobic digestion, which can produce biogas to generate electricity. Bioremediation, animal feed, bio-stimulants, and biopesticides are other useful applications (28).

### OMWW for health supplements and cosmetics products

OMWW has been found to possess antioxidant and anti-microbial properties, making it a potential ingredient in health supplements and cosmetic products. Extracting valuable compounds from OMWW presents an opportunity to obtain sustainable ingredients for the production of functional and fortified foods. For instance, phenolic rich extracts from OMWW have been satisfactorily added to an olive spread (29) and to breadsticks (30). Supplementary Table 1 shows the polyphenol content of the batches of the OMWW extract, named A009, which was characterized and studied by our group.

The A009 phenol rich purified extract used by our team was obtained from Massimo and Daniele Pizzichini according to Patent formulation (Patent US 8,815,815 B2). Initially, a ceramic microfiltration (MF) was conducted using 2 tubular membranes made of alumina oxide with a 300 KDa cut-off (TAMI membranes, Nyons, France) and a filtration surface area of 0.35 m2. This step effectively removed solid particles, residual plant matter, and cells, all of which were subsequently discarded. The resulting MF permeate was then subjected to further concentration through reverse osmosis (RO) using a Polyamide spiral wound module (Microdyn Nadir, Wiesbaden, Germany) with a surface area of 7 m^2^. The RO permeate was essentially purified water and was also discarded. Ultimately, the RO concentrate, achieved at a volume concentration ratio (VCR) of 3.6, constituted the olive extract, A009.

Today, a lot of consumers are interested in supplements containing vitamins, minerals, and other nutrients, and more than ever, research-based data is needed to properly advise customers, particularly about natural ingredients. A substantial obstacle for “clean label” ingredients has been created at the same time by the growing interest in the components used in food items.

A study revealed that when compared to the control, a fortified juice (as OliPhenolia®) with the addition of the phenols concentrate from OMWW did not exhibit off-flavor or off-odor. Additionally, the supplemented juice showed a stable phenol concentration after 60 days of refrigeration, results that could be applied in the production of orange juice that has a natural antioxidant concentration added as a “clean label” ingredient (31). In another investigation, Foti et al. (32) sought to produce a novel functional beverage beginning with OMWW that would have a health-promoting effect. The fermentation of OMWW, utilizing microbial pools in both single- and co-cultures resulted in an increase in HT and TYR concentrations as compared to the control sample. Fermented OMWW might be suggested as a new beverage and/or functional component that may also contain compounds as flavorings and probiotic microbes (32).

The potential for creating a novel nutraceutical product based on olive pâté (OP) and OMWW was investigated in a different study. In order to fulfill the European community’s claims about a potential antioxidant effect on plasma lipids, researchers were able to produce a product that was high in trans resveratrol, OH tyrosol, and tyrosol. From both a commercial and nutraceutical point of view, the product had a promising market outlook due to its good palatability and stable results (29).

Cosmeceuticals are another interesting area of application for OMWW extracts. The strong antioxidant, anti-microbial, and anti-inflammatory properties of the OMWW fraction A009 offer protection from skin diseases (Table 1), improved skin health and beneficial effects on the skin ageing process (33, 42). An investigation of a biophenols extract, derived from an upcycling strategy using leftovers from the olive agri-food industry (from *Olea europaea* leaves and waste water), was prepared and tested as a cosmetic product and a dietary supplement (35). A considerable and progressive improvement in the state of the skin was seen after the combined action of the cosmetic and food supplement formulation, on 46 healthy volunteers, in a period of 8 weeks (35). This improvement was attributed to increases in collagen content, skin elasticity, and skin hydration (skin health indicators). Additionally, the therapy reduced the irritating effects of chemical agents and UV rays by acting as a skin protector (35). Drying OMWW polyphenols using a spray drying technique on human keratinocytes (HaCaT cell model) has been shown to improve cell repair and migration in scratch assays (43). Furthermore, a pro-oxidative and pro-apoptotic effect of a polyphenolic OMWW fraction on the UVA-damaged HEKa keratinocyte cells was observed (34). A009 has shown a potential to improve and prolong hair growth *in vivo*, and due to its antioxidant properties, it may help to maintain a healthy scalp and perhaps stop hair loss brought on by oxidative stress (36) (Table 1). OMWW’s potential extends to the nutraceutical ophthalmic domain, where it could be utilized against various inflammatory conditions affecting the ocular surface. This is attributed to the presence of free radical scavengers in its composition. *In vitro* studies have already positioned OMWW for upscaling in ophthalmic nutraceutical applications (44). Several studies have suggested that the phenolic compounds in OMWW may have beneficial effects on cardiovascular health. For example, HT has been shown to reduce blood pressure and improve endothelial function, which could help to prevent atherosclerosis and other cardiovascular diseases (45–48). Hara et al. (41) investigated the effect of OMWW and HT on atherogenesis, OMWW (0.30% w/w) or HT (0.02% w/w) were added to a western-type diet for 20 weeks and fed to male apolipoprotein E-deficient mice. Without affecting body weight, plasma cholesterol levels, or blood pressure, OMWW and HT slowed the progression of atherosclerosis in the aortic arch, in a comparable manner. The aorta’s generation of oxidative stress, as well as the expression of inflammatory molecules like IL-1 and MCP-1 and NADPH oxidase subunits like NOX2 and p22phox, were all reduced by OMWW and HT. (41)

**Table 1:** Effects of OMWW extracts on normal cells.

	OMWW extract		
Cells	*In vitro*	*In vivo*	References
Keratinocyte	Antibacterial effect against Gram-negative and Gram-positive bacteria. Antioxidant effect reducing ROS formation. Anti-inflammatory effect, reducing IL-8. Photoprotection UVA-damaged human keratinocytes	Improvement of skin hydration and collagen density, enhancement of skin elasticity and decrease of erythema index.	(33) (34) (35)
Human follicle dermal papilla	Positive influence on cell proliferation and release of growth factors IGF-1. Antioxidant effect reducing ROS formation and preventing oxidative stress.	Help in improving and extending hair growth.	(36)
Endothelial (HUVEC)	Suppression of proliferation, increase of apoptosis, inhibition of endothelial morphogenesis, migration, and invasion.	Inhibition of the angiogenic response induced by VTH: (VEGF, TNF-α, Heparin).	(37)
Cardiomyocytes	Enhancement of the proliferative-reducing effect of 5-FU and cisplatin. Reduction of IL-6 mRNA induced by 5-FU.	Reduction of mitochondria damage induced by chemotherapy cisplatin, 5-FU or doxorubicin	(38) (39)
Skeletal muscles		Functional amelioration of oxidative stress in 27-month-old rats	(40)
Aorta		Decrease of atherogenesis, IL-1β, CXCR2 levels, reduction of oxidative genes NOX2 and p22phox in Apolipoprotein E-deficient male mice fed on western-type diet.	(41)

The OMWW extract has positive effects on normal cell lines, both *in vitro* and *in vivo*, which are summarized in the table.

A study showed that an HT enriched OMWW extract exerts a potent antioxidant and significant anti-microbial activity against two olive tree pathogens (*Pseudomonas savastanoi pv. savastanoi* and *Agrobacterium tumefaciens*) (49). Against therapeutically relevant Gram-positive and Gram-negative infections, resistant and multi-resistant to current antibiotic drugs, various OMWW samples demonstrated considerable antibacterial activity (50, 51).

The increased phenolic levels or other ingredients present in the mixture, such as fatty acids, may be the cause of the enhanced antibacterial properties (52).

A recent review reported studies with bioactive compounds from olive by-products, that confirm ingesting olive-derived products promotes health. But little research has been done so far and further human studies are needed to confirm safety and health-promoting properties of olive oil by-products (53). An *in vivo* study showed for the first time that using OMWW as dietary supplementation can prevent cell death and tissue deterioration, and the harmful effects of oxidative stress in the cellular systems of rabbits (54).

In summary, due to its anti-inflammatory, antioxidant, and anti-microbial properties, both *in vitro* and *in vivo* evidence indicated the prospective use of OMWW as a food supplement and in skin cosmetic products.

### OMWW effect on normal and tumor associated endothelial cells

A promising and perhaps less investigated aspect is the anti-angiogenic and angio-preventive potential of phenolic compounds. Angiogenesis is the process of forming new blood vessels from existing ones (55–58).

Under normal bodily conditions, cell multiplication is tightly regulated to balance programmed cell death (apoptosis), maintaining tissue size. However, tissue growth, like during increased metabolic demands, relies on angiogenesis. In healthy adults, angiogenesis is typically restrained, except during specific events like the female reproductive cycle (for endometrial regeneration and corpus luteum formation), pregnancy (for placenta development), and wound healing (granulation tissue formation) (55–59). Anti-angiogenesis is valuable in contexts like cancer treatment, where it curtails pathologic blood vessel growth, restricting tumor blood supply and inhibiting tumor growth (56, 57, 59). Achieving this balance between inhibiting pathological angiogenesis, often seen in diseases like cancer, and promoting physiological angiogenesis, crucial for tissue repair and development, is a complex challenge. This task involves targeted strategies that interrupt abnormal blood vessel growth in disease while enhancing functional vessel formation where necessary. One effective approach is targeting specific molecules or receptors overexpressed during pathological angiogenesis, such as growth factor (GF), GF receptors or integrins, effectively blocking the formation of abnormal blood vessels by inhibiting vascular endothelial growth factor (VEGF) signaling (55–59).

Our group carried out a study to evaluate the anti-angiogenic and angio-preventive potential of A009 on HUVECs proliferation, induction of apoptosis, migration, and network formation activities *in vitro*, and its ability to interfere with angiogenesis both *in vitro* and *in vivo* (37). To make sure that activity was not affected by potential changes in chemical composition and compounds present in the extract, such as those influenced by seasonal weather variations and the amount of time between olive oil extraction and extract preparation, experiments were conducted using two different batches of A009. HT, used as control, was from synthetic origin and with a purity of ≥98%. The anti-proliferative potential of A009 was evaluated by testing its ability to hinder endothelial cell proliferation, and then compared to the effect of HT alone at a similar concentration (Table 1).

A009 displayed the ability to suppress HUVEC proliferation after 24 h (dilution 1:1000), and at higher concentrations it completely arrested it (37). In contrast, HT alone, at a similar dilution, exhibited a comparatively lower effect. These results suggest that the presence of a diverse mixture of phenolics in the A009 extract enhances its anti-proliferative activity. The team then examined whether the cytostatic impact of A009 was linked to the induction of apoptosis, by performing a flow cytometry-based apoptosis assay. After 48 h of treatment, HUVECs showed increased apoptosis with A009 as compared to HT alone (37).

A009 was also shown to inhibit reactive oxygen species (ROS) production before and after H_2_O_2_ treatment (37). When there is an imbalance between the production of ROS and the antioxidant capacity of the host, it results in oxidative stress. ROS are generated as by-products during the mitochondrial electron transport of aerobic respiration, or by oxidoreductase enzymes and metal-catalyzed oxidations and are associated with several inflammatory conditions. Given the antioxidant compounds contained in A009, the group investigated its ability to scavenge ROS in HUVECs as compared to HT alone. A009 exhibited robust ROS scavenger effects in both pre- and post-treatment. Conversely, HT alone exerted little ROS scavenger activity (37).

In another study, the OMWW extracts showed antioxidant activity protecting both HUVECs and human pulmonary artery smooth muscle cells from oxidative stress-induced cell death (60).

*In vitro*, A009 can inhibit endothelial morphogenesis (37). When adding pro-angiogenic factors to endothelial cells plated on Matrigel, a reconstituted basement membrane matrix, HUVECs can form capillary-like networks (61, 62). A study showed that A009 was able to interfere with HUVECs morphogenesis in a dose-dependent manner, at a similar level to HT alone. A009 can also inhibit HUVECs migration and invasion. To form new blood vessels during angiogenesis, endothelial cells need to traverse basement membranes. Hence, the team’s investigation focused on determining whether A009 and HT could impact the migration and invasion ability of HUVECs through Matrigel. A009 showed a significant decrease in the number of migrated and invaded endothelial cells in a dose-dependent manner, at dilutions of 1:500 and 1:250. Purified HT also exhibited a significant inhibitory effect on migration and invasion at the highest concentration, although to a lower extent (Table 1).

Additionally, the inhibitory effect of A009 on angiogenesis was assessed *in vivo* (37). The study investigated the effect of A009 and HT on *in vivo* angiogenesis using a subcutaneous Matrigel sponge assay. The results showed that A009 inhibited the angiogenic response induced by a pro-angiogenic cocktail (VTH: VEGF, TNF-α, heparin), as detected by macroscopic inspection, and quantified by Drabkin’s assay for haemoglobin. HT showed limited effects on angiogenesis (Table 1). The inhibition of angiogenesis by A009 was confirmed by histological examination of the Matrigel pellets (37).

Bender et al. (63, 64) evaluated the bioavailability of HT and its metabolites, contained in food supplements obtained from OMWW (OliPhenolia bitter®, OliPhenolia®), in a randomized and controlled human trial. Bioavailability is a prerequisite for any compound effect, and they observed that after the ingestion of the food supplement, HT is absorbed and highly metabolized into its metabolites, which are likely the primary contributors to the positive effects observed (63, 64). In light of these findings, OMWW holds great promise as a food supplement for the prevention of oxidative stress *in vivo* and the resulting cardiovascular risk (63), as well as in preventing lipoxidation (64).

Exercise-induced aerobic metabolism increase is a well-known potential source of oxidative stress. Athletes typically use supplements made of plant-derived polyphenols to improve performance, hasten the recovery of muscle function, and lessen the adverse effects of exercise-induced oxidative stress (65, 66). Roberts and colleagues investigated the effect of OMWW (OliPhenolia®) on aerobic workout and acute recovery in healthy volunteers. They observed a modest antioxidant effect following the intake (16 days) of OMWW food supplements, rich in HT, with suppression of superoxide dismutase (SOD) activity and increased glutathione (GSH) in the 24 h after a 60 min intense aerobic exercise session (65, 66). An *in vivo* study demonstrated that treatment of 27-month-old rats with OMWW enriched in HT successfully alleviated skeletal muscle function decline originating from age-related oxidative stress (40).

There is some evidence to suggest that the phenolic compounds in OMWW may help to improve insulin sensitivity and regulate blood sugar levels, which could be beneficial for people with diabetes (45–48). Phenolic compounds in OMWW may have neuroprotective effects and could help to prevent or slow the progression of neurodegenerative diseases such as Alzheimer’s (67–69) and Parkinson’s (69). The effectiveness of a HT-rich OMWW extract to lessen Fe^2 + −^ and nitric oxide (NO)-induced cytotoxicity in murine-dissociated brain cells was examined due to the detrimental effect that oxidative stress has on brain cell survival (70). Oral long-term HT consumption has the potential to protect against numerous oxidative stress pathways, according to *in vivo* and *ex vivo* findings (70). In TgCRND8 mice (double-mutant gene of APP695) it was observed that the effects of oleuropein aglycone on behavioral performance and neuropathology are not closely related to oleuropein aglycone by itself, in fact, a comparable neuroprotective effect was observed using a diet supplementation with the same dose of a mix of polyphenols found in the OMWW (71).

In summary, all the discussed studies provide evidence that bioactive compounds in OMWW can be valid allies in the treatment of various pathologies, with particular effect on angiogenesis, supporting the strong pharma-nutritional potential in cardiovascular, neurological and oxidative stress disorders.

### Olive oil by-products and OMWW against cancer

OMWW extracts have shown promising results as candidates to prevention and treatment of cancer. Phytochemicals are particularly appealing as cancer chemopreventive agents due to their low toxicity and ability to modulate various signal transduction pathways in biological processes associated with cancer (72). Chemoprevention refers to the administration of bioactive molecules to block, revert, or delay the carcinogenic process. Chemopreventive agents can reduce cancer risk in several ways, such as proliferation inhibition, apoptosis induction, and angiogenesis inhibition. In recent years, studies have suggested that applying well-tolerated dietary supplements, such as carotenoids, green tea catechin, curcumin, fish oil fatty acids, and polyphenols, can reduce the risk of cancer development or progression through their antioxidant, anti-proliferative, anti-angiogenic, anti-inflammatory and pro-apoptotic effects in various types of cancers. HT and TYR, two main components of EVOO and OMWW, have been particularly associated with anti-proliferative and pro-apoptotic effects. The numerous biological properties of HT have been demonstrated *in vitro* and *in vivo* by a number of studies (73–77) and have also been recognized by the European Food Safety Authority (EFSA).

In cancer, angiogenesis plays a crucial role in tumor growth and metastasis. Indeed, tumors require a blood supply to provide oxygen and nutrients necessary for their growth and survival. While in normal tissue, angiogenesis is tightly regulated, in cancer, the balance between pro- and anti-angiogenic factors is disrupted, leading to the development of new blood vessels that feed the tumor (55–58). Cancer cells produce factors that promote angiogenesis, such as vascular endothelial growth factor (VEGF) and many others for review (56–58, 78), which stimulate the growth of new blood vessels. Anti-angiogenic therapy for cancer aims to inhibit the formation of new blood vessels and starve the tumor of its blood supply. This can be achieved by using drugs that target VEGF, CXCL8 or other pro-angiogenic factors, or their receptors. The ability of the OMWW extract A009 to affect cell proliferation and survival has been evaluated on colon, lung, prostate, breast, bladder, and melanoma human tumor cell lines (Table 2). Both functional and *in vivo* studies showed that A009 was able to inhibit tumor cell line growth in a dose dependent manner, showing a comparatively stronger inhibitory effect than HT alone.

**Table 2:** Effect of OMWW extract on cancer cells.

	OMWW extract		
Cells	*In vitro*	*In vivo*	Combination with chemotherapy
Colon cancer	Suppression of proliferation, apoptosis, migration, invasion, adhesion, sprouting. Downregulation of VEGF and IL-8 (79). Negative regulation of NF-κB phosphorylation and TNF-α levels. Increase of PPARγ (80).	Slower growth of tumor mass (39).	Enhancement of the cisplatin and 5-FU drugs effect (39).
Lung cancer	Suppression of proliferation, induction of apoptosis, limitation of cell migration and invasion. Reduction in pro-angiogenic factors. Downregulation of CXCR4 and CXCL12 expressions (81).		
Prostate cancer	Reduction of cell viability, adhesion, migration, invasion, sprouting, and colonies formation (82).	Reduction of tumor size (39).	Enhancement of the cisplatin drug effect (39).
Breast cancer	Reduction of cell growth. Reduction size of breast cancer cell spheroids in combination with chemotherapy drugs (38).	Reduction of angiogenesis and enhancement of the T cell immune cell number (38).	Enhancement of the cisplatin, doxorubicin and 5-FU drugs effect (38).
Bladder cancer	Inhibition of growth and proliferation, both in chemo-sensitive and gemcitabine- and cisplatin-resistant tumour cells (83).		
Melanoma	Inhibition of A375 melanoma nodules growth in the melanoma skin model (33).		

The table lists the OMWW extract’s effect on several cancer cell types, both *in vivo* and *in vitro*, as well as in combination with currently available chemotherapeutic drugs.

Another olive tree by-product, leaf extracts, also exhibited anti-cancer and anti-inflammatory properties (84–87). These activities, in particular, are related to oleuropein, a main chemical compound of olive leaves. A recent study has demonstrated that oleuropein-rich leaf extracts (named ORLE), exert anti-tumor and anti-inflammatory activities in colon tumors, reducing cell proliferation and increasing cell apoptosis. Moreover, it is able to reduce nitric oxide synthase (iNOS) in colon tumor lesions and peritoneal macrophages of Apc-mutated PIRC rats (85). The extract helps to inhibit the pro-inflammatory signal generated by cancer cells or inflammatory cells of the tumor microenvironment, which is essential for the progression of colon cancer. Based on their results, the researchers suggest ORLE as a complementary therapy in combination with standard anti-cancer drugs (85).

Other studies have shown that the oil leaf extract has anti-proliferative and pro-apoptotic activity on both triple-negative breast and ovarian cancer cells (86). It is also effective on melanoma, reducing growth and inhibiting metastatic spread (87).

A diet rich in polyphenols, such as those found in olive oil and the Mediterranean diet or a by-product of olive oil production, can reduce the risk of developing colon cancer. Our study investigated the potential chemopreventive properties of A009 on colorectal cancer (CRC) cell lines (79). We used a murine xenograft model to test the effect of A009 on the growth of CT-26 CRC cells, using purified HT as a control. The results showed that A009 inhibited the proliferation, migration, invasion, adhesion, and sprouting of CRC cells, and also decreased the release of pro-angiogenic and pro-inflammatory cytokines (VEGF, IL-8) to a similar extent to HT alone. Moreover, *in vivo* experiments showed that A009 was more effective than HT alone in slowing down the growth of CT-26 tumors (79). The A009 enhanced the effect of the cisplatin and 5-fluorouracil (5-FU), two common chemotherapeutic drugs, on HT29 CRC cells (39). In human CRC-derived cell lines (HCT116 and LoVo) HT and the OMWW extracts decrease proliferation, normal (colonic CCD-841CoN; skin fibroblast WS1) cells are less responsive (80), OMWW extracts (88) increased apoptosis in CRC-derived cell lines. OMWW extracts negatively regulated NF-κB phosphorylation as TNF-α and IL-8, and PPARγ levels increase (80). OMWW extracts and HT promoted mitochondrial functionality involving the PPARγ/PGC-1α axis in HCT116 and LoVo CRC cells (89). Cardinali et al. (89) studied an OMWW fraction rich in verbascoside and isoverbascoside, estimating their bioavailability both in *in vitro* digestion and Caco-2 human intestinal cell models. In the *in vitro* model to assess the bioaccessibility of phenolic compounds from OMWW, digestive recoveries were found to be 35.5% ± 0.55% for verbascoside and 9.2% ± 0.94% for isoverbascoside, underscoring the potential sensitivity of these phenolics to gastric and small intestine digestive conditions. Uptake of verbascoside and isoverbascoside was rapid, with peak accumulation occurring after 30 min, providing a rationale for subsequent *in vivo* studies on the bioavailability and bioactivity of OMWW components (89). In human colorectal carcinoma cells, an OMWW extract showed antioxidant effects against ileo-carcinoma cell line HCT8 cells modulating the intracellular ROS content (90).

Despite progress in targeted therapies, lung cancer remains the leading cause of cancer death worldwide. While avoidance of smoking is the most effective measure, chemoprevention could be useful, particularly for high-risk individuals. Our group evaluated the chemopreventive effects of A009 extracts on lung cancer cell lines (A549 and H1650) (81). A009 extracts inhibited cell proliferation, induced apoptosis, limited cell migration and invasion, and decreased the production of pro-angiogenic factors. The work demonstrated that the A009 extracts inhibited the growth of A549 and H1650 lung cancer cells in a time- and dose-dependent manner, and this was linked with increased apoptosis at 24 and 48 h, with higher induction in H1650 compared to A549 cells. A009 extracts also reduced the production of CXCR4 and CXCL12, which regulate cell migration and invasion, and inhibited the formation of invasive sprouts on Matrigel. Additionally, A009 extracts interfered with the production of pro-angiogenic factors, including VEGF, CXCL8, and CCL2, in both cell lines (81).

A009 can exert chemopreventive activities also for prostate cancer (PCa). Cell lines (PC-3, DU-145, LNCaP) were tested *in vitro* (82). Surface-Activated Chemical Ionization/Electrospray Ionization mass spectrometry (SACI/ESI-MS) was used to determine the polyphenol content in the extracts. The mass spectrometry analysis confirmed HT as the major component of A009, which was used as a reference compound to test the OMWW extract’s chemopreventive properties *in vitro*. A009’s chemopreventive activity was tested in proliferation assays and functional studies for cell adhesion, migration, and invasion. It was found to significantly reduce PCa cell viability up to 96 h in all cell lines investigated, similarly to HT. A009 inhibited PCa cell adhesion, migration, invasion, and sprouting, including interfering with LNCaP cell line’s ability to form colonies/islets *in vitro* (82). Furthermore, A009 was able to inhibit VEGF production and CXCL8 release in all PCa cell lines investigated, and angiogenin only in LNCaP cells (82). *In vivo*, the A009 extract co-administered with cisplatin showed a synergistic effect in further reducing tumor size in mouse xenografts of PCa (39).

In a further study we investigated the anti-cancer effects of A009 on breast cancer cells (MDA-MB-231 and BT-549) when combined with chemotherapeutic agents such as doxorubicin, an anthracycline commonly used in breast cancer therapy, and 5-FU as the prototype fluoropyrimidine. *In vitro* experiments showed that A009 combined with doxorubicin or 5-FU effectively decreased breast cancer cell growth, and additive effects were observed in breast tumor spheroids (38). A009 was anti-angiogenic in the Matrigel sponge model containing breast cancer cells supernatants, and recruitment of T cells was increased by A009 (38).

Bladder cancer is a threatening tumor of the urinary system, approximately 90% of all bladder cancers worldwide are urothelial carcinoma. OMWW injection solution (Burg-Apotheke) was tested on resistant parental bladder cancer cell lines (T24, TCCSUP, and RT112) for 48 and 72 h proliferation (83). The clonogenic cell growth, number and size were significantly diminished in a concentration dependent manner. Cisplatin-resistant and gemcitabine-resistant tumor cells were exposed to OMWW, and their growth resulted significantly diminished. The OMWW treatment reduced pAkt (24 and 72 h), pRaptor (24 h), Rictor (24 and 72 h), pRictor, and pmTOR Akt–mTOR axis. It is assumed that OMWW’s ability to inhibit growth and proliferation, both in chemosensitive and gemcitabine- and cisplatin-resistant bladder cancer cells, is associated with cell cycling arrest through cyclin-CDK axis manipulation and suppression of Akt/mTOR pathway activation (83).

OMWW was also able to modulate the invasion of tumor cells in a 3D melanoma skin model, affecting both the growth and migration of melanoma cells and the melanoma nodes formation (33). A375 cells, human metastatic melanoma cells, were used and the treatment with A009 reduced A375 melanoma nodules growth *in vitro* compared to the untreated samples as determined by cell cluster size reduction (33).

The data collected in this review showed that olive oil industry by-products and their main bioactive compounds inhibited pro-inflammatory cytokines, as well as other molecules involved in inflammatory processes and in several cancer types as potential adjuvant therapy. Molecular targets that A009 extract decreased in several cancer cells are shown in Table 3.

**Table 3:** Molecular targets reduced by OMWW extract in cancer cells.

Cancer Cells	Molecular target	References
Colon	VEGF CXCL8 NF-kB TNFα Increase PPARγ	(79) (80)
Lung	CXCR4 CXCL12 VEGF CXCL8 CCL2	(81)
Prostate	VEGF CXCL8 Angiogenin	(82)
Bladder	pAkt pRaptor pRictor pmTOR Akt–mTOR axis	(83)

### OMWW as cardioprotective during anti-cancer therapy

One of the most undesirable side effects in cancer patients receiving chemotherapy is cardiovascular toxicity, which can limit the effectiveness of treatment options (91–93), including combinations of different anti-cancer agents (38, 39). Chemotherapy drugs, commonly used in several neoplasms, are known for their cardiotoxicity (38, 39). A cardioprotective role was observed in mice co-treated with A009 extracts alongside cisplatin; the hearts of these mice showed reduced mitochondria damage compared to those treated with chemotherapy alone (38). *In vitro* we observed toxicity on rat cardiomyocyte H9C2 cells treated with chemotherapy drugs (doxorubicin, cisplatin, and 5-FU), while cell proliferation was not affected by A009 alone. However, co-treatment with A009 and either cisplatin or 5-FU did not further reduce cardiac cell proliferation induced by chemotherapy (39). When A009 was combined with 5-FU, it showed cardioprotective effects on neonatal murine cardiomyocytes. Co-treatment resulted in a smaller reduction in the number of cardiomyocytes compared to treatment with 5-FU alone (39). Additionally, A009 demonstrated cardioprotective effects in zebrafish embryos. Co-treatment with A009 reversed the doxorubicin-induced cardiotoxic effect, particularly in terms of cardiac area. Furthermore, it exhibited the ability to reduce the upregulation of the pro-inflammatory IL-6 and p16 mRNA induced by 5-FU in human cardiomyocytes. These findings indicate its potential as a preventative agent in cardio-oncology (39). Inhibition of cancer cells was extensive while effects on cardiomyocytes were limited (Table 1).

Cardiovascular toxicities are still one of the complications of chemotherapy (91–93). The studies indicate that the OMWW purified extract could exert cardiovascular protection (Table 1).

All of the above studies suggest that the polyphenol-rich OMWW extract is highly effective. Its polyphenols are more abundant and less expensive than purified single components, and the compounds can be assumed orally as a nutraceutical (61). Not only does A009 appear effective in preventing the growth of several tumor cells, but it can also prepare cells to respond better to chemotherapy (Table 1).

Mitochondrial damage is a key cause of cardio-toxicities brought on by chemotherapy because cardiomyocytes heavily rely on them for their energy needs. Polyphenols counteract the production of ROS that causes cellular and mitochondrial damage by acting as antioxidants.

Cardioprotective effect is reported in an ultrastructural analysis, mice treated with A009 extracts and cisplatin together showed less mitochondrial damage, displaying a rounder form, and having more, better-organized mitochondrial cristae, than mice treated with chemotherapy alone. Additionally, it was shown that animals treated with A009 and cisplatin together had more regular dispositions of muscular myosin and actin fibers in their hearts than animals treated with cisplatin alone. Finally, only hearts treated with cisplatin had inflammation and fibrosis visible under optical microscopy (39).

These data show that the use of OMWW extract could be a promising strategy to reduce chemotherapy-induced cardiotoxicity in oncological patients, influencing effect on the distribution of muscle proteins and mitochondrial function (39).

Scarce data about the OMWW mechanisms of action are found in the literature, so further investigation focused on fully understanding the mechanisms underlying OMWW cardioprotective effects is needed.

### Environmental issues of OMWW

The circular economy is a system focused on reducing waste and promoting sustainability by prolonging the use of resources. It was included among the 17 United Nation Sustainable Development Goals adopted by all the Member States in 2015. These goals aim to globally implement strategies that enhance health and education, reduce inequality, stimulate economic growth, all while addressing climate change and preserving our oceans and forests.^1^

It is a model that aims to create a closed-loop system where materials, products, and waste are continually reused, recycled, or repurposed, instead of being discarded. Food by-products are the leftover materials generated during the production, processing, and consumption of food. These by-products can include vegetable peels, fruit scraps, and animal bones, among others. In an enhancement of the circular economy, food by-products can be transformed into valuable resources, rather than being treated as industry waste. This can be achieved through a range of approaches, including:

- Upcycling: transforming food by-products into new products with a higher value, such as turning fruit waste into juices, jams, or supplements.
- Recycling: converting food by-products into new materials or products, such as compost or biofuel.
- Reusing: finding alternative uses for food by-products, such as using vegetable peels in animal feed.

Recycling olive oil by-products as nutraceuticals is a very promising proposition (43, 50, 94–97). The olive tree perhaps really is the first of all trees (“Olea prima omnium arborum est” is its Latin epithet), and EVOO, together with its powerful watery companion, OMWW, possibly are the “liquid gold” often mentioned throughout history.

## Conclusion

The review emphasizes how crucial it is to recover phenolic compounds from OMWW and other by-products so that they can be used in the food and nutriceutical industries while also making a sustainable contribution to waste treatment. The findings from our studies and those of other research teams hold significant promise for the potential applications of purified extracts from both olive mill waste water and olive leaf extracts in the field of cancer prevention and treatment. The observed capacity of these extracts to not only reduce cancer cell growth but also to induce apoptosis and inhibit angiogenesis highlights their multifaceted potential as candidates for combating cancer. These attributes make them attractive prospects for further exploration in the realm of anti-cancer supplements.

Moreover, it is noteworthy that olive mill waste water extracts have demonstrated the ability to enhance the effectiveness of chemotherapeutic drugs while protecting the heart and cardiomyocytes. This synergy suggests that these extracts could serve as valuable adjuncts to conventional cancer treatments, potentially leading to more efficacious less toxic therapies.

Our studies reveal that while these extracts exhibit potent effects against cancer cells, they appear to have a comparatively lesser impact on cardiomyocytes. This selective action suggests a potential safety profile that could be advantageous in clinical applications.

Beyond their biomedical potential, the utilization of waste products from agricultural processing, such as these olive by-products extracts, carries a positive environmental impact. By repurposing what would otherwise be discarded, research into the utilization of olive oil by-products aligns with sustainable practices and contributes to the reduction of waste in the agricultural sector.

However, it is important to emphasize that while these initial findings are promising, further research is imperative. A comprehensive understanding of the underlying mechanisms behind the effects of olive production by-products is needed. This knowledge will enable the development of more targeted and effective strategies for their utilization in diverse fields, including nutraceuticals, cosmeceuticals, and cancer preventive extracts. This underscores the significance of continued investigations in order to unlock the full potential of these natural and processed compounds for the betterment of human health and the environment.

## Author contributions

AA: Conceptualization, Writing – original draft. FA: Writing – original draft. PC: Writing – review & editing. LD: Writing – review & editing. LC: Writing – review & editing. DN: Conceptualization, Writing – original draft.

## Footnotes

^1^ https://sdgs.un.org/goals
