# Supplementary material for: From antiquity to contemporary times: how olive oil by-products and waste water can contribute to health
Source: Front Nutr. 2023 Oct 16;10:1254947. doi: 10.3389/fnut.2023.1254947 (PMC10615083; doi:10.3389/fnut.2023.1254947)
Supplement: Supplementary file 1 [file Table_1.DOCX]

**Table S1.** Phenolic content in OMWW (A009) extract. Quantification of the phenolic presence in A009 batches was performed using high performance liquid chromatography (HPLC) analysis. Results are expressed as g/L. (ND: Not Detected).

|  | **A009** | | | |
| --- | --- | --- | --- | --- |
| **PHENOLIC COMPOUND** | **BATCH A**  **(g/L)** | **BATCH B**  **(g/L)** | **BATCH C**  **(g/L)** | **BATCH D**  **(g/L)** |
| **Hydroxytyrosol glucoside** | ND | 1.69 | 1.91 | 2.01 |
| **Hydroxytyrosol** | 2.7 | 5.72 | 5.50 | 2.52 |
| **Tyrosol** | 0.2 | ND | 0.69 | 0.80 |
| **Chlorogenic acid** | 0.12 | 0.10 | 0.13 | 0.10 |
| ***β*-Hydroxyverbascoside isomer 1** | 0.35 | 0.14 | 0.23 | 0.29 |
| ***β*-Hydroxyverbascoside isomer 2** | 0.32 | 0.17 | 0.23 | 0.26 |
| **Verbascoside** | 0.84 | 1.32 | 1.07 | 1.04 |
| **Caffeoyl ester of secologanoside** | ND | 0.20 | 0.23 | 0.66 |
| **Oleouropein aglycon** | ND | 0.22 | 0.21 | 0.71 |
| **6’-*p*-Coumaroyl secologanoside** | ND | 0.40 | 0.35 | 0.38 |
| **Rutin** | 0.11 | ND | ND | ND |
| **Luteolin-7-*o*-glucoside** | 0.22 | ND | ND | ND |
